# Cancer Cachexia in Advanced Renal Cell Carcinoma: From Molecular Mechanisms to Prognostic Assessment

**DOI:** 10.3390/ijms27177944

**Published:** 2026-09-06

**Authors:** Yushuang Cui, Yudong Cao, Chen Lin, Jinchao Ma, Shuo Wang, Peng Du

**Affiliations:** Key Laboratory of Carcinogenesis and Translational Research (Ministry of Education), Department of Urology, Peking University Cancer Hospital, Beijing 100142, China

**Keywords:** cancer cachexia, renal cell carcinoma, sarcopenia, systemic inflammation, prognostic assessment, immunotherapy

## Abstract

Cancer cachexia is a multifactorial metabolic syndrome characterized by progressive skeletal muscle loss, affecting 30–60% of patients with advanced renal cell carcinoma (RCC). It significantly impacts treatment tolerance, quality of life, and prognosis, yet its diagnosis and management remain challenging due to fragmented RCC-specific evidence, particularly in the era of immune checkpoint inhibitor (ICI)-based therapy. The pathogenesis involves persistent systemic inflammation, metabolic reprogramming, and tumor–host interactions. The IL-6/STAT3 and TNF-α/NF-κB pathways are central to muscle catabolism, while tumor-derived mediators such as GDF15 and PTHrP, along with mitochondrial dysfunction, further drive cachexia progression. For prognostic assessment, CT-derived skeletal muscle mass evaluation combined with systemic inflammatory and nutritional biomarkers—including neutrophil-to-lymphocyte ratio (NLR), modified Glasgow Prognostic Score (mGPS), prognostic nutritional index (PNI), and cachexia index (CXI)—has improved risk stratification in advanced RCC. Preclinical and emerging clinical data suggest that targeted therapies may partially attenuate cachexia by modulating inflammatory signaling, while multimodal interventions integrating nutritional support and exercise rehabilitation remain the cornerstone of management. Novel strategies, such as inhibition of the GDF15/GFRAL axis, are under active investigation. Future research should prioritize identification of early biomarkers, standardization of cachexia assessment, and prospective evaluation of cachexia-directed interventions in the immunotherapy era. Integrating cachexia assessment into routine practice may ultimately enable personalized treatment and improve long-term outcomes for patients with advanced RCC.

## 1. Introduction

Renal cell carcinoma (RCC) is the most common malignancy of the kidney, accounting for approximately 2–3% of all adult cancers [1]. Although the introduction of targeted therapies and immune checkpoint inhibitors (ICIs) has substantially improved the treatment landscape of advanced RCC, metastatic RCC (mRCC) remains associated with poor long-term outcomes, with a 5-year overall survival rate of less than 15% [1]. Consequently, identifying factors beyond tumor burden that influence treatment response and survival has become an important focus of contemporary RCC research.

Cancer cachexia is a complex multifactorial metabolic syndrome characterized by ongoing loss of skeletal muscle mass, with or without adipose tissue depletion, that cannot be completely reversed by conventional nutritional support and leads to progressive functional impairment [2,3]. According to the international consensus proposed by Fearon et al. in 2011, cancer cachexia encompasses a spectrum ranging from pre-cachexia to refractory cachexia [4]. More recently, the 2023 Asian Working Group for Cachexia (AWGC) updated the diagnostic criteria for Asian populations, placing greater emphasis on sarcopenia and objective assessment of muscle function. These advances have shifted the clinical evaluation of cachexia from body weight alone toward a more comprehensive assessment of body composition and physical performance [5].

Cachexia affects approximately 30–60% of patients with advanced RCC and has emerged as an important determinant of treatment tolerance, quality of life, and survival [1]. Previous studies have demonstrated that early skeletal muscle loss during targeted therapy is independently associated with poor clinical outcomes [6,7], while the coexistence of sarcopenia and systemic inflammation further worsens prognosis [8,9]. With the widespread adoption of ICI-based combination therapies as first-line treatment for advanced RCC, increasing attention has been directed toward understanding how cachexia influences treatment efficacy, immune responses, and long-term clinical outcomes [10,11].

The pathogenesis of cancer cachexia is highly complex and involves dynamic interactions among systemic inflammation, metabolic reprogramming, neuroendocrine alterations, tumor-derived catabolic factors, and host immune responses. In RCC, inflammatory mediators such as interleukin-6 (IL-6) and tumor necrosis factor-α (TNF-α) activate the STAT3 and NF-κB signaling pathways, promoting ubiquitin–proteasome-mediated skeletal muscle protein degradation [12,13,14]. Emerging evidence further implicates tumor-derived mediators, including growth differentiation factor 15 (GDF15) and parathyroid hormone-related protein (PTHrP) [15,16,17], as well as mitochondrial dysfunction and extracellular vesicle-mediated intercellular communication, in the development and progression of cachexia [18].

Accurate assessment of cachexia is essential for individualized management of patients with advanced RCC. Conventional anthropometric measures such as body mass index (BMI) inadequately reflect skeletal muscle depletion, whereas computed tomography (CT)-derived skeletal muscle index (SMI), psoas muscle index (PMI), and systemic inflammatory and nutritional biomarkers—including the C-reactive protein/albumin ratio (CAR), neutrophil-to-lymphocyte ratio (NLR), prognostic nutritional index (PNI) [19,20,21], and the cachexia index (CXI)—have demonstrated superior value for prognostic stratification [10]. Increasingly, multimodal assessment integrating body composition, nutritional status, and systemic inflammation has been recognized as a more comprehensive approach for evaluating cachexia severity and predicting clinical outcomes.

Despite increasing recognition of the clinical significance of cancer cachexia, several important challenges remain in patients with advanced RCC. Current evidence is derived predominantly from retrospective studies with heterogeneous diagnostic criteria, and many mechanistic insights originate from pan-cancer investigations rather than RCC-specific research. Moreover, the biological interactions between cachexia and contemporary ICI-based therapies, the optimal biomarkers for early diagnosis, and evidence-based management strategies have yet to be fully established. These knowledge gaps highlight the need for an updated and disease-specific synthesis of current evidence.

In this review, we comprehensively summarize recent advances in the definition and diagnosis of cancer cachexia, discuss the molecular mechanisms underlying cachexia in advanced RCC, evaluate the prognostic significance of cachexia-related biomarkers, and review current and emerging therapeutic strategies. Particular emphasis is placed on recent evidence generated in the era of immune checkpoint inhibitor-based therapy, with the aim of providing an evidence-based framework for future translational research and multidisciplinary clinical management of patients with advanced RCC.

## 2. Overview of Cancer Cachexia

### 2.1. Definition and Diagnostic Criteria of Cancer Cachexia

Cancer cachexia has long been recognized as a complex metabolic syndrome, yet its definition and diagnostic framework have evolved substantially over the past decades. The most widely adopted diagnostic criteria were established by Fearon et al. in the 2011 international consensus, which defined cancer cachexia as “a multifactorial syndrome characterized by an ongoing loss of skeletal muscle mass (with or without loss of fat mass) that cannot be fully reversed by conventional nutritional support and leads to progressive functional impairment” [4]. Importantly, this consensus introduced a three-stage clinical model comprising pre-cachexia, cachexia, and refractory cachexia, reflecting the progressive nature of the syndrome. According to these criteria, cachexia is diagnosed by involuntary weight loss exceeding 5% within six months, or by weight loss greater than 2% in individuals with a BMI below 20 kg/m^2^ or established sarcopenia [4]. More than a decade later, the Fearon consensus remains the conceptual foundation for both clinical practice and cancer cachexia research.

However, increasing clinical evidence has highlighted several limitations of the original framework. Although body weight loss is a convenient and clinically accessible indicator, it does not adequately reflect changes in skeletal muscle mass or function. This limitation is particularly relevant in patients with RCC, in whom substantial muscle depletion may occur before clinically apparent weight loss, potentially resulting in delayed recognition of cachexia. In addition, the BMI threshold proposed in the Fearon criteria may be less appropriate for Asian populations, whose baseline body composition differs considerably from that of Western populations. These shortcomings have prompted efforts to refine cachexia assessment by incorporating direct measurements of muscle quantity and physical function.

To address the limitations of previous frameworks, the Asian Working Group for Cachexia (AWGC) updated the diagnostic criteria for Asian populations in 2023. This update represents a significant conceptual shift in the diagnostic paradigm for cancer cachexia. In contrast to the 2011 Fearon criteria, which incorporated reduced skeletal muscle mass as one of the core diagnostic pathways, the AWGC 2023 guidelines deliberately excluded direct muscle mass assessment from the mandatory diagnostic criteria [5]. Rather than emphasizing structural sarcopenia, the AWGC framework pivots toward a multidimensional evaluation of systemic inflammation and physical function. Specifically, the mandatory parameters now include C-reactive protein (CRP > 5 mg/L) as a biomarker of inflammation and handgrip strength (cut-offs: <28 kg for men, <18 kg for women) as a measure of muscle function, alongside a weight loss criterion of >2% over 3–6 months and a BMI threshold of <21 kg/m^2^. Consequently, this conceptual evolution moves the diagnostic focus toward a functionally oriented definition of cachexia, which is particularly relevant for patients with advanced RCC where inflammation and functional decline are predominant clinical features. By integrating functional performance with inflammatory biomarkers, the AWGC criteria provide a more population-specific framework that may improve the early identification and staging of cachexia in Asian patients with cancer.

Alongside the evolution of diagnostic criteria, advances in body composition imaging have substantially improved the objective assessment of cachexia. CT-derived measurements, particularly the SMI and PMI at the third lumbar vertebra (L3), have become the most widely used surrogate markers for skeletal muscle mass in oncology [19,22]. Numerous studies have demonstrated that CT-defined sarcopenia is closely associated with adverse clinical outcomes and provides greater prognostic value than conventional anthropometric measurements in patients with advanced RCC [9]. Other assessment modalities, including dual-energy X-ray absorptiometry (DXA), bioelectrical impedance analysis (BIA), and ultrasonography, also contribute to the evaluation of muscle mass and body composition, although their clinical applicability varies according to availability and measurement standardization [20].

Taken together, the concept of cancer cachexia has evolved from a diagnosis primarily based on body weight loss to a multidimensional syndrome encompassing skeletal muscle quantity, muscle function, nutritional status, and systemic metabolic alterations. For patients with advanced RCC, this transition is particularly important because early identification of muscle wasting may facilitate more accurate prognostic stratification and provide opportunities for timely therapeutic intervention. The major differences between the Fearon consensus and the AWGC 2023 [5] criteria are summarized in Table 1.

### 2.2. Epidemiology and Clinical Significance

The prevalence of cancer cachexia varies substantially according to tumor type and disease stage, affecting approximately 50–80% of patients with advanced malignancies [1]. Among solid tumors, pancreatic, gastric, and lung cancers exhibit the highest incidence of cachexia, whereas approximately 30–60% of patients with advanced RCC develop this syndrome [1,12]. Notably, considerable interindividual variability exists even among patients with the same tumor type and stage, suggesting that both tumor-specific biological characteristics and host-related factors contribute to the initiation and progression of cachexia.

In patients with advanced RCC, the development of cachexia is closely associated with tumor burden, metastatic status, and treatment modality. A systematic review and meta-analysis demonstrated that preoperative sarcopenia is an independent predictor of poor postoperative survival in patients undergoing surgery for RCC [23]. Similarly, a study focusing on sarcomatoid RCC reported significantly higher rates of postoperative complications and cancer-specific mortality among patients with preoperative sarcopenia [22]. These findings indicate that cachexia is not merely a manifestation of advanced disease but also an important determinant of postoperative recovery and long-term oncological outcomes.

Beyond its prognostic significance, cachexia substantially influences treatment tolerance in advanced RCC. Accumulating evidence suggests that baseline skeletal muscle mass and nutritional status are associated with the occurrence of dose-limiting toxicities during systemic therapy [3,24]. Patients with low BMI or CT-defined sarcopenia receiving tyrosine kinase inhibitors (TKIs), such as sorafenib or sunitinib, are more likely to experience severe treatment-related adverse events, dose reductions, or premature treatment discontinuation. These observations highlight the clinical relevance of body composition assessment before initiating systemic therapy and support its incorporation into individualized treatment planning.

With the widespread adoption of ICI-based combination therapies as the standard first-line treatment for advanced RCC, increasing attention has been directed toward the interaction between cachexia and antitumor immunity [25,26]. Emerging evidence suggests that cachexia may influence treatment efficacy through multiple mechanisms, including impaired nutritional status, chronic systemic inflammation, and alterations in immune function. Although the precise biological interactions remain incompletely understood, these findings underscore the need to further investigate cachexia within the context of modern immunotherapy.

Importantly, the clinical consequences of cachexia extend beyond survival outcomes. Progressive skeletal muscle wasting contributes to fatigue, reduced physical performance, impaired functional independence, and diminished quality of life, which in turn adversely affect treatment adherence and social participation. This vicious cycle may further accelerate physical decline and compromise the overall effectiveness of anticancer therapy. Therefore, early identification and multidisciplinary management of cachexia should be considered an integral component of comprehensive care for patients with advanced RCC [27].

Taken together, current evidence indicates that cancer cachexia represents a clinically significant systemic complication rather than simply a consequence of tumor progression. Its close associations with postoperative outcomes, treatment tolerance, quality of life, and survival emphasize the importance of incorporating cachexia assessment into routine clinical evaluation. These observations also provide the rationale for exploring the biological mechanisms underlying RCC-associated cachexia, which are discussed in the following section.

## 3. Pathophysiological Mechanisms of Cancer Cachexia in Advanced Renal Cell Carcinoma

### 3.1. Systemic Inflammation and Metabolic Reprogramming

Persistent low-grade systemic inflammation is widely recognized as a central driver of cancer cachexia. Rather than representing a simple consequence of tumor progression, chronic inflammation actively orchestrates the metabolic alterations that underlie skeletal muscle wasting and energy imbalance. In patients with advanced RCC, tumor-associated immune dysregulation results in sustained elevation of multiple pro-inflammatory cytokines, thereby establishing a systemic catabolic environment. Early experimental studies using RCC cell lines demonstrated increased production of TNF-α, interleukin-1β (IL-1β), and IL-6, which was subsequently confirmed by studies showing enhanced cytokine production by peripheral blood mononuclear cells from patients with RCC [14,28].

Among these inflammatory mediators, IL-6 has been the most extensively investigated in the context of cancer cachexia. Elevated IL-6 expression in clear cell RCC (ccRCC) is associated with reduced skeletal muscle mass and inferior survival outcomes [29]. Mechanistically, IL-6 binds to its receptor complex containing gp130 and activates the JAK/STAT3 signaling pathway, which serves as a pivotal regulator of muscle protein catabolism. Persistent activation of STAT3 induces the transcription of the E3 ubiquitin ligases muscle RING finger-1 (MuRF-1) and muscle atrophy F-box (MAFbx), thereby accelerating ubiquitin–proteasome-mediated degradation of myofibrillar proteins [12]. Experimental studies further demonstrated that pharmacological inhibition of STAT3 not only suppresses tumor growth but also significantly attenuates skeletal muscle wasting in RCC-bearing mice, highlighting STAT3 as a potential molecular link between tumor progression and cachexia [13].

TNF-α represents another key inflammatory mediator involved in RCC-associated cachexia. Increased circulating TNF-α levels correlate with weight loss and systemic inflammatory markers, including C-reactive protein (CRP), in patients with RCC [30]. Binding of TNF-α to tumor necrosis factor receptor 1 (TNFR1) activates the NF-κB signaling pathway, which further amplifies inflammatory responses and promotes muscle protein degradation. In addition to stimulating the ubiquitin–proteasome system, NF-κB suppresses the myogenic transcription factor MyoD, thereby impairing skeletal muscle regeneration. TNF-α also contributes to insulin resistance and enhanced lipolysis, further exacerbating systemic metabolic disturbances.

Inflammation and metabolic reprogramming are closely interconnected processes during the development of cancer cachexia. Similar to many malignancies, RCC cells undergo metabolic reprogramming characterized by enhanced aerobic glycolysis (the Warburg effect), enabling sustained tumor growth while increasing host energy expenditure [31]. Consequently, patients develop insulin resistance, enhanced gluconeogenesis, and progressive depletion of energy reserves. Simultaneously, accelerated lipolysis in white adipose tissue and increased thermogenesis in brown adipose tissue further aggravate the negative energy balance characteristic of cachexia. Emerging evidence also implicates mitochondrial dysfunction as an important contributor to metabolic remodeling. Tumor-derived extracellular vesicles have been shown to alter mitochondrial dynamics and bioenergetics in skeletal muscle and adipose tissue, thereby amplifying tissue wasting and systemic metabolic dysfunction [18].

The systemic inflammatory response is further reflected by acute-phase reactants, among which CRP is the most widely used clinical biomarker. Beyond serving as an indicator of inflammatory burden, elevated CRP may also contribute to the persistent catabolic state through interactions with complement activation and immune regulation [8,9]. Accordingly, inflammation-based prognostic models such as the mGPS, which incorporates CRP and serum albumin levels, have been validated as independent predictors of postoperative and survival outcomes in patients with RCC receiving surgery or targeted therapy [32,33].

Collectively, current evidence suggests that chronic inflammation and metabolic reprogramming constitute a mutually reinforcing biological network that drives the initiation and progression of cancer cachexia in advanced RCC. The central roles of the IL-6/STAT3 and TNF-α/NF-κB signaling pathways also provide a strong biological rationale for developing anti-inflammatory and metabolism-targeted therapeutic strategies, which are discussed in subsequent sections.

### 3.2. Tumor-Derived Factors and Tumor–Host Interactions

Tumor-derived soluble mediators play a direct and indispensable role in the development of cancer cachexia by establishing a complex network of communication between tumor cells and host tissues. Beyond the classical inflammatory cytokines discussed above, increasing evidence indicates that multiple tumor-derived factors directly regulate appetite, energy metabolism, skeletal muscle homeostasis, and adipose tissue remodeling. Rather than acting independently, these mediators interact with systemic inflammation and metabolic reprogramming to accelerate the progression of cachexia.

Among these mediators, GDF15 has emerged as one of the most extensively investigated molecules. GDF15, a member of the transforming growth factor-β (TGF-β) superfamily, is highly expressed in various malignancies, including RCC [15,16]. By binding to the glial cell-derived neurotrophic factor family receptor α-like (GFRAL) in the area postrema of the brainstem, GDF15 suppresses appetite and promotes body weight loss through central regulation of energy balance. Beyond its role in cachexia, elevated circulating GDF15 levels have also been associated with clinical responses to immune checkpoint inhibitors, suggesting that GDF15 may represent a mechanistic link between metabolic dysfunction and antitumor immunity in RCC [16].

Another important tumor-derived mediator is PTHrP. Experimental studies have demonstrated that PTHrP promotes the browning of white adipose tissue and enhances thermogenesis, thereby increasing whole-body energy expenditure and contributing to progressive weight loss [17]. Although the molecular mechanisms of PTHrP in RCC-associated cachexia remain incompletely understood, available evidence suggests that similar metabolic alterations are present in patients with advanced RCC, highlighting PTHrP as another potential therapeutic target.

At the cellular level, skeletal muscle wasting is primarily executed through activation of intracellular protein degradation pathways. The ubiquitin–proteasome system (UPS) represents the principal mechanism responsible for muscle protein breakdown. Activation of the NF-κB and STAT3 signaling pathways induces the expression of the E3 ubiquitin ligases MuRF-1 and atrogin-1, thereby accelerating ubiquitination and degradation of contractile proteins such as myosin heavy chain and actin [12]. In parallel, dysregulated autophagy–lysosome activity further contributes to muscle atrophy. While physiological autophagy is essential for maintaining cellular homeostasis through the removal of damaged proteins and organelles, excessive or persistent autophagic activation during cachexia promotes progressive loss of muscle fibers [13]. Furthermore, activation of apoptotic pathways, characterized by increased Caspase-3 activity and an altered Bax/Bcl-2 ratio, may further exacerbate skeletal muscle depletion.

Host immune responses further shape the progression of cachexia through reciprocal interactions with tumor-derived mediators. Alterations in T-cell function and expansion of regulatory T cells (Tregs) may indirectly influence muscle wasting by modifying the systemic cytokine milieu. In addition, changes in Fc receptor expression within the tumor microenvironment have been proposed to influence the pharmacokinetics and therapeutic efficacy of anticancer agents, thereby potentially affecting treatment responses in cachectic patients [34]. These observations suggest that cachexia should be regarded not only as a metabolic disorder but also as an immunometabolic syndrome involving dynamic interactions between tumor cells and the host immune system.

Emerging evidence has further expanded the biological landscape of cancer cachexia. Gut microbiota dysbiosis has been implicated in regulating systemic inflammation, nutrient metabolism, and skeletal muscle homeostasis, although its specific contribution to RCC-associated cachexia remains largely unexplored. Likewise, chemerin, an adipokine involved in immune and metabolic regulation, has recently been reported to influence skeletal muscle phenotype in patients with kidney disease [35]. Although evidence in RCC remains limited, these findings provide new insights into the interaction between renal dysfunction and cancer-associated muscle wasting and may identify novel therapeutic targets for future investigation.

**Figure 1:**
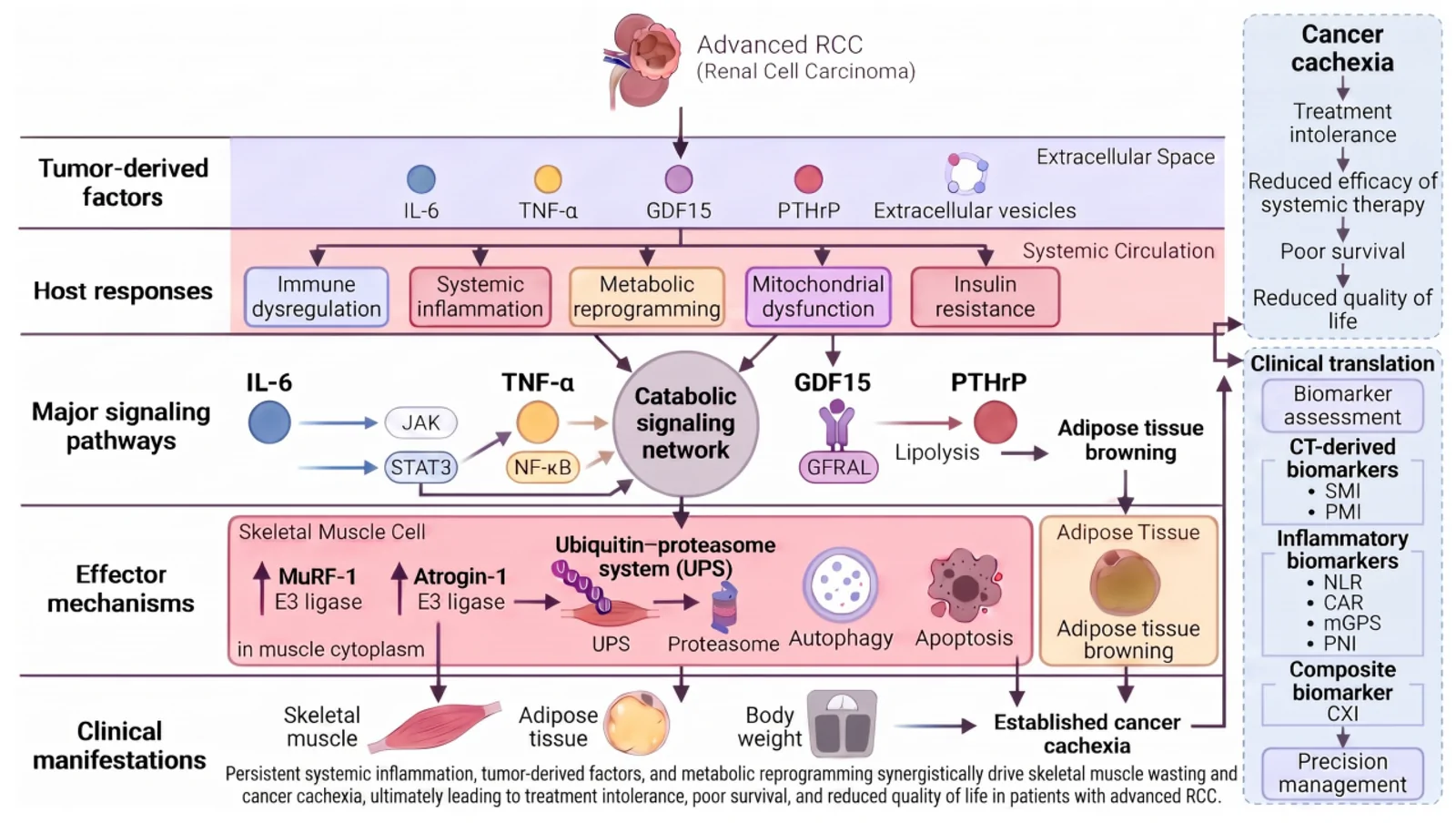
Molecular mechanisms underlying cancer cachexia in advanced RCC. Tumor-derived mediators, including IL-6, TNF-α, GDF15, PTHrP, and extracellular vesicles, induce systemic inflammation, metabolic reprogramming, mitochondrial dysfunction, and immune dysregulation. These alterations activate major catabolic pathways such as IL-6/JAK/STAT3 and TNF-α/NF-κB signaling, leading to ubiquitin–proteasome activation, autophagy, apoptosis, skeletal muscle wasting, adipose tissue browning, and ultimately cancer cachexia. The resulting syndrome contributes to treatment intolerance, poor survival, and reduced quality of life while providing potential opportunities for biomarker-guided precision management.

Overall, tumor-derived factors and host responses constitute an integrated biological network that cooperatively drives the development of cancer cachexia in advanced RCC. While inflammatory cytokines initiate systemic catabolism, tumor-derived mediators, intracellular protein degradation pathways, and immune dysregulation collectively sustain muscle wasting and metabolic dysfunction. A better understanding of these interconnected mechanisms may facilitate the identification of novel biomarkers and targeted therapies for cachexia in patients with advanced RCC. The major molecular pathways and their interactions involved in RCC-associated cachexia are summarized in Figure 1.

### 3.3. Cachexia-Related Biomarkers and Prognostic Assessment

Accurate assessment of cancer cachexia has evolved from simple body weight measurements to a multidimensional evaluation integrating body composition, systemic inflammation, and nutritional status. Among these approaches, CT-based assessment of skeletal muscle mass has become the most objective and widely adopted method for evaluating cachexia in patients with advanced RCC.

CT-derived SMI and PMI, typically measured at the level of the L3, have consistently demonstrated prognostic value in both localized and metastatic RCC [19,22]. Multiple retrospective and prospective studies have identified low baseline SMI as an independent predictor of shorter overall survival in patients receiving targeted therapy or ICI-based treatment [6,36]. Compared with conventional anthropometric measurements, CT-derived body composition provides a more accurate representation of skeletal muscle depletion and therefore enables improved risk stratification.

Importantly, longitudinal changes in skeletal muscle mass may provide greater prognostic information than a single baseline assessment. In patients with mRCC receiving first-line sunitinib, early skeletal muscle loss during the initial 6–8 weeks of treatment independently predicted subsequent overall survival [7,37]. Similar findings have been reported in patients treated with nivolumab, in whom both baseline sarcopenia and progressive muscle loss during treatment were associated with inferior clinical outcomes [38]. These observations suggest that serial assessment of body composition may facilitate early identification of patients at increased risk of treatment failure and disease progression.

Beyond imaging-based evaluation, systemic inflammatory biomarkers represent another important component of cachexia assessment. The NLR and platelet-to-lymphocyte ratio (PLR) have been associated with an increased risk of sarcopenia and adverse clinical outcomes [39]. Likewise, the CAR and the mGPS integrate inflammatory and nutritional status and have demonstrated prognostic value in patients with RCC undergoing surgery or systemic therapy [32,33]. The PNI, calculated from serum albumin concentration and peripheral lymphocyte count, has also been validated as an independent predictor of overall survival in advanced RCC [21,40].

Recognizing that cancer cachexia is a multifactorial syndrome, recent studies have increasingly focused on integrated prognostic models rather than individual biomarkers. Among these, the CXI combines skeletal muscle mass, systemic inflammation, and nutritional status into a single composite indicator. CXI has demonstrated favorable prognostic performance in patients with metastatic RCC receiving nivolumab plus ipilimumab and appears to outperform individual physiological parameters for predicting treatment response and survival [10,11]. Similarly, CT-based radiomics models are emerging as promising non-invasive tools for characterizing tumor biology and improving prognostic prediction in RCC [41].

Several additional biomarkers have also shown potential clinical utility. Circulating GDF15, particularly when combined with IL-6, has been investigated as a predictor of immunotherapy response [16]. Nutritional assessment tools such as the Controlling Nutritional Status (CONUT) score have likewise demonstrated prognostic value in patients receiving targeted therapy [21]. Furthermore, recent studies suggest that both baseline adipose tissue composition and longitudinal changes in body fat measured by CT may provide complementary prognostic information in patients treated with ICIs [25,26,42].

Although the International Metastatic RCC Database Consortium (IMDC) risk model remains the most widely used prognostic system for advanced RCC, it does not incorporate body composition or cachexia-related parameters. Increasing evidence suggests that integrating CT-defined sarcopenia and systemic inflammatory biomarkers into existing prognostic models may improve risk discrimination and facilitate more individualized treatment strategies [8,43].

**Figure 2:**
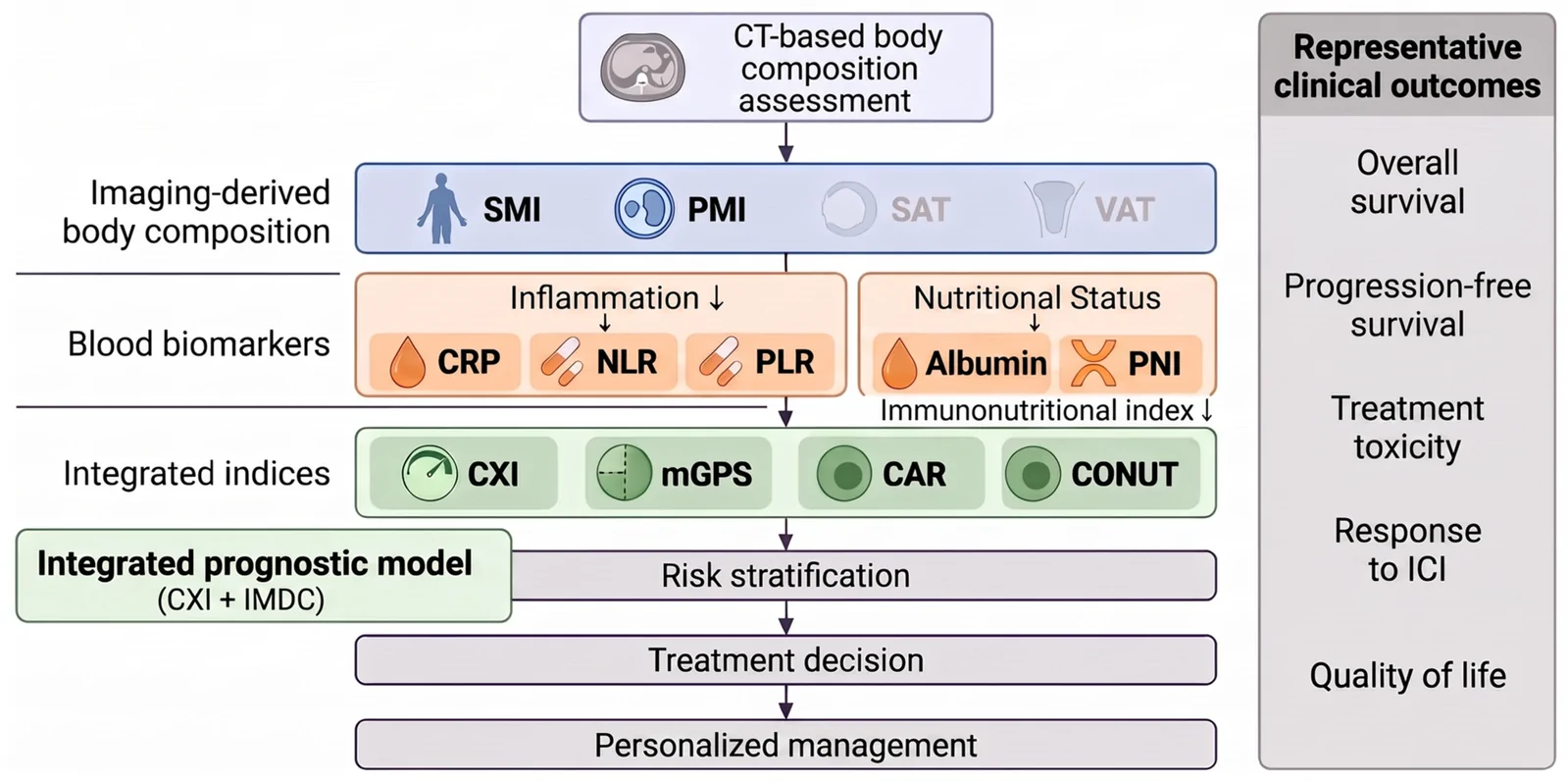
Multidimensional assessment and prognostic framework for cancer cachexia in advanced renal cell carcinoma.

Overall, current evidence indicates that no single biomarker adequately captures the complexity of cancer cachexia. Instead, multimodal assessment integrating body composition, systemic inflammation, nutritional status, and emerging imaging biomarkers appears to provide the most comprehensive evaluation of prognosis in patients with advanced RCC. Future studies should focus on prospective validation of these integrated models and their incorporation into routine clinical decision-making, particularly in the era of immunotherapy. Cachexia-related biomarkers and their clinical significance are summarized in Table 2, whereas representative translational evidence supporting cachexia assessment and management is presented in Table 3. An integrated framework for multidimensional prognostic assessment is illustrated in Figure 2.

CT-derived body composition (SMI and PMI), inflammatory biomarkers (CRP, NLR, and PLR), nutritional indicators (albumin and PNI), and composite prognostic indices (CXI, mGPS, CAR, and CONUT) are integrated to improve risk stratification, guide treatment decisions, and facilitate personalized management. These multidimensional assessments are associated with overall survival, progression-free survival, treatment toxicity, response to immune checkpoint inhibitors, and quality of life. The proposed framework provides a practical strategy for integrating cachexia assessment into routine clinical decision-making in advanced RCC.

## 4. Treatment Strategies for Cancer Cachexia in Advanced RCC

**Figure 3:**
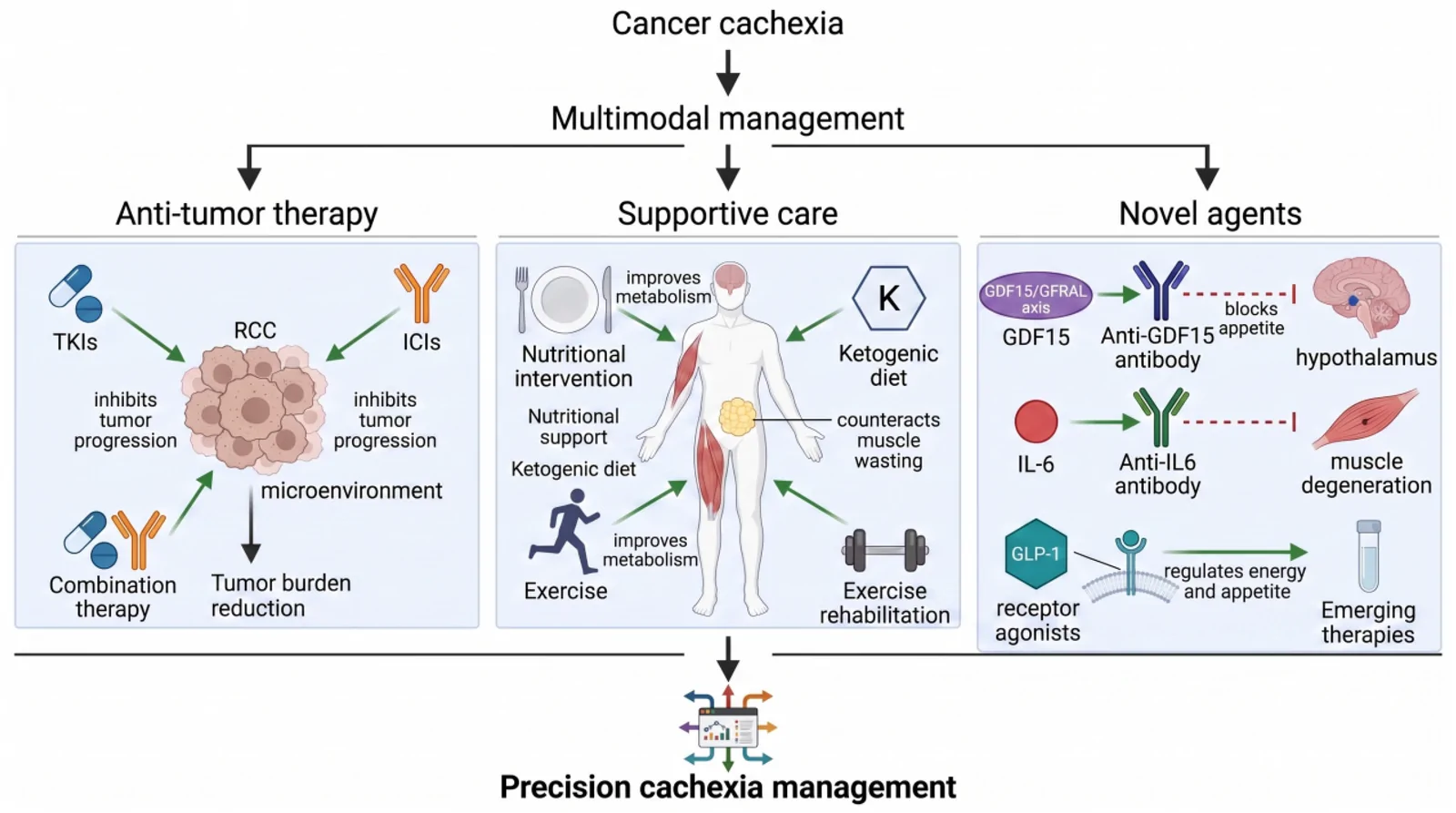
Current and emerging strategies for the management of cancer cachexia in advanced RCC. Multimodal treatment combines effective antitumor therapy (TKIs, immune checkpoint inhibitors, and combination therapy), supportive interventions (nutritional support, ketogenic diet, exercise, and rehabilitation), and novel targeted approaches including GDF15/GFRAL blockade, IL-6 inhibition, and GLP-1 receptor agonists. Integration of these approaches aims to achieve precision cachexia management and improve clinical outcomes.

The management of cancer cachexia requires a multidisciplinary and multimodal approach, as no single intervention can fully reverse the complex metabolic alterations underlying this syndrome. Effective management should therefore integrate optimal anticancer therapy with nutritional support, exercise rehabilitation, symptom control, and, when appropriate, targeted pharmacological therapy. The current multimodal management strategy for cancer cachexia in advanced RCC is summarized in Figure 3.

### 4.1. Anticancer Therapy as the Foundation of Cachexia Management

Because persistent tumor activity is a major driver of systemic inflammation and metabolic dysregulation, effective anticancer treatment remains the cornerstone of cachexia management. Preclinical studies have demonstrated that sunitinib not only inhibits tumor growth but also attenuates skeletal muscle wasting by suppressing the STAT3/MuRF-1 signaling pathway in RCC-bearing mice [12]. Clinical studies further suggest that patients whose skeletal muscle mass remains stable or improves during sunitinib treatment experience superior survival outcomes [7,37]. Similarly, cabozantinib, a multi-target tyrosine kinase inhibitor directed against MET, VEGFR2, and AXL, has demonstrated encouraging therapeutic activity in patients with mRCC accompanied by sarcopenia [36]. Although current evidence primarily originates from retrospective analyses, these findings suggest that certain targeted therapies may exert dual benefits by simultaneously controlling tumor progression and mitigating cachexia. With ICI-based combination therapy becoming the standard first-line treatment for advanced RCC, increasing attention has focused on the interaction between cachexia and antitumor immunity. On one hand, successful immunotherapy may indirectly improve cachexia by reducing tumor burden and systemic inflammatory activity. On the other hand, immune-related inflammatory responses and immune-mediated toxicities may aggravate skeletal muscle loss in susceptible patients [25,38].

Furthermore, baseline sarcopenia has been proposed to influence the pharmacokinetics and treatment exposure of ICIs, potentially contributing to heterogeneous therapeutic responses [34,45]. Although the underlying mechanisms remain incompletely understood, these observations highlight the importance of evaluating body composition before initiating immunotherapy and monitoring muscle mass longitudinally during treatment. Such an approach may facilitate individualized treatment optimization and early supportive intervention.

### 4.2. Nutritional Support and Exercise Rehabilitation

Nutritional intervention and structured exercise remain the cornerstone of supportive care for patients with cancer cachexia. A recent systematic review and meta-analysis demonstrated that combined nutritional supplementation and exercise rehabilitation improve physical function and attenuate muscle wasting in patients with chronic disease-related cachexia [27]. Within the RCC setting, a pilot study suggested that a ketogenic diet may be feasible and safe during systemic treatment for advanced RCC [46]. Under professional nutritional supervision, ketogenic dietary intervention may help maintain metabolic homeostasis and reduce treatment-related weight loss. However, current evidence remains preliminary, and adequately powered randomized controlled trials are required before routine clinical implementation can be recommended.

### 4.3. Emerging Targeted Therapies and Personalized Cachexia Management

Growing understanding of the molecular mechanisms underlying cachexia has stimulated the development of mechanism-based therapeutic strategies. Although no pharmacologic agent is currently approved specifically for cancer cachexia in RCC, emerging agents targeting distinct pathogenic nodes have shown promise in advanced malignancies and may be relevant in the RCC setting. Anamorelin, a ghrelin-receptor agonist, significantly increased lean body mass and body weight in two phase 3 trials (ROMANA 1 and ROMANA 2) involving patients with advanced NSCLC and cachexia, although handgrip strength was not improved [47,48]. Ponsegromab, a humanized monoclonal antibody against GDF-15, was evaluated in the phase 2 PROACC-1 trial (NCT05546476) in patients with cancer cachexia and elevated GDF-15 (≥1500 pg/mL); ponsegromab treatment resulted in dose-dependent weight gain (median between-group difference of 1.22, 1.92, and 2.81 kg at 100, 200, and 400 mg, respectively) and improvements in appetite, cachexia symptoms, and physical activity [49,50]. These findings establish GDF-15 as a validated therapeutic target in cachexia. Likewise, GLP-1 receptor agonists, owing to their metabolic regulatory properties, have attracted increasing interest as potential adjunctive therapies, particularly in combination with immunotherapy [51,52]. Real-world studies in ICI-treated patients with lung cancer and overweight/obesity reported lower all-cause mortality (HR 0.65), fewer hospitalizations, and a lower incidence of cachexia-related outcomes with GLP-1 RA use, although residual confounding precludes causal inference [51]. However, prospective evidence specific to RCC-associated cachexia remains limited.

Regarding anti-cytokine therapies, preclinical and early clinical evidence supports targeting the inflammatory cascade [53]. In a randomized phase II trial of advanced pancreatic cancer, the addition of the IL-6 receptor antibody tocilizumab to gemcitabine/nab-paclitaxel reduced muscle loss (median change +0.10% vs. −3.43% at 2 months, *p* = 0.0012) and improved overall survival at 18 months, although the primary endpoint was not met and treatment-related adverse events were increased [54]. Likewise, clazakizumab (ALD518) improved anemia, attenuated lean body mass loss, and reversed fatigue in patients with NSCLC [55]. Conversely, agents targeting TNF-α (e.g., infliximab, etanercept) and IL-1α (e.g., MABp1) have yielded mixed results, and broader phase III validation remains lacking [56,57]. IL-1 receptor antagonism with anakinra has demonstrated anti-cachectic effects in preclinical models [58], but prospective clinical evidence in cancer cachexia is still limited. While early findings from these malignancies are encouraging, the efficacy and safety of IL-6 and IL-1 blockade in RCC-associated cachexia require further investigation through prospective clinical trials.

For patients with refractory cachexia, treatment goals should shift from reversing muscle loss to optimizing symptom control, preserving functional independence, and improving quality of life. Appetite stimulants, corticosteroids, and comprehensive palliative care remain important components of supportive management. Looking forward, cancer cachexia management is expected to evolve toward a precision medicine paradigm. Rather than relying on isolated interventions, future strategies will likely combine effective tumor control with individualized nutritional support, exercise rehabilitation, anti-inflammatory therapy, and metabolism-targeted agents. Early identification of high-risk patients using integrated biomarkers may further enable timely intervention before irreversible muscle wasting develops.

Overall, current evidence supports a multimodal strategy as the optimal approach for managing cancer cachexia in advanced RCC. However, most available interventions are supported by limited prospective evidence, and robust randomized clinical trials are still needed to determine whether cachexia-directed therapies can improve survival, treatment tolerance, and quality of life in the immunotherapy era.

## 5. Conclusions

Cancer cachexia has become increasingly recognized as a major systemic complication of advanced RCC, substantially affecting treatment tolerance, quality of life, and survival. Rather than representing a simple consequence of tumor progression, cachexia is now understood as a multifactorial syndrome driven by chronic systemic inflammation, tumor-derived catabolic factors, metabolic reprogramming, and host immune responses. Advances in diagnostic criteria and body composition assessment have shifted clinical evaluation from body weight alone to multidimensional assessment incorporating skeletal muscle mass, muscle function, nutritional status, and inflammatory biomarkers. Meanwhile, CT-based muscle measurements and integrated biomarkers such as the CXI have improved prognostic stratification, while increasing mechanistic insights have identified several promising therapeutic targets. Although multimodal interventions integrating anticancer therapy, nutritional support, exercise rehabilitation, and emerging targeted therapies appear promising, their clinical benefits require further validation.

Despite substantial progress, several important challenges remain. Early identification of cachexia, standardization of diagnostic approaches, and prospective validation of cachexia-directed interventions are needed, particularly in patients receiving ICI-based therapies. Future research should emphasize mechanism-based precision management by integrating imaging, circulating biomarkers, and individualized therapeutic strategies. A better understanding of tumor–host interactions and earlier intervention may ultimately facilitate personalized care and improve long-term outcomes for patients with advanced RCC.

## Figures and Tables

**Table 1:** Comparison of the Fearon (2011) [4] and AWGC (2023) [5] diagnostic criteria for cancer cachexia.

Criteria	Fearon 2011 [4]	AWGC 2023 [5]	Clinical Significance
Weight loss	>5% involuntary loss	≥2% over 3–6 months	Initial indicator of cachexia progression
BMI	<20 kg/m^2^	<21 kg/m^2^ (Asian-specific)	Ethnic-specific nutritional risk assessment
Skeletal muscle	Suggested (CT/DXA)	Optional/auxiliary	Prognostic/auxiliary assessment
Muscle function	Not included	Grip strength (<28 kg men, <18 kg women) and/or gait speed	Functional assessment
Inflammation (CRP)	Not included	Required (>5 mg/L)	Prognostic/auxiliary assessment
Cachexia stage	Pre-cachexia → Cachexia → Refractory	Similar staging	Guides disease management

Abbreviations: BMI, body mass index; DXA, dual-energy X-ray absorptiometry; AWGC, Asian Working Group for Cachexia.

**Table 2:** Imaging, inflammatory, nutritional, and composite biomarkers for prognostic assessment of cancer cachexia in advanced renal cell carcinoma.

Biomarker	Category	Biological Significance	Clinical Application
SMI	CT-derived	Skeletal muscle mass	Prognostic assessment
PMI	CT-derived	Muscle reserve	Prognostic assessment
NLR	Inflammatory	Systemic inflammation	Survival prediction
PLR	Inflammatory	Inflammation	Risk assessment
CAR	Composite	CRP + Albumin	Prognostic stratification
mGPS	Composite	CRP + Albumin	Prognostic model
PNI	Nutritional	Nutrition + immunity	Treatment tolerance
CONUT	Nutritional	Nutritional status	Prognostic assessment
CXI	Composite	Muscle + inflammation + nutrition	Precision stratification

Abbreviations: SMI, skeletal muscle index; PMI, psoas muscle index; NLR, neutrophil-to-lymphocyte ratio; PLR, platelet-to-lymphocyte ratio; CAR, C-reactive protein-to-albumin ratio; mGPS, modified Glasgow Prognostic Score; PNI, prognostic nutritional index; CONUT, Controlling Nutritional Status; CXI, cachexia index.

**Table 3:** Representative clinical evidence supporting the assessment and management of cancer cachexia in advanced renal cell carcinoma.

Category	Representative Evidence	Clinical Implication	Future Direction
CT-derived body composition	Antoun et al. [6,36]	Predicts poor survival	Prospective validation
	Martin L et al. [44]	Predicts postoperative outcomes	Standardized CT cut-offs
	Meta-analysis [19]	Confirms prognostic value of sarcopenia	Large prospective studies
Inflammatory and nutritional biomarkers	mGPS, CAR, PNI, CONUT [17,28,29,39]	Improve risk stratification	Dynamic monitoring
	CXI (Yamashita et al.) [10,11]	Predicts survival and ICI response	External validation
Therapeutic strategies	TKIs (sunitinib/cabozantinib) [6,35,36]	Preserve muscle and improve outcomes	Cachexia-guided treatment.
	Ketogenic diet [44]	Feasible supportive strategy	Randomized trials
	GDF15/GFRAL, IL-6-targeted therapy [30,31,45,46]	Emerging therapeutic targets	RCC-specific validation

Abbreviations: CT, computed tomography; ICI, immune checkpoint inhibitor; TKI, tyrosine kinase inhibitor; RCC, renal cell carcinoma; mGPS, modified Glasgow Prognostic Score; CAR, C-reactive protein-to-albumin ratio; PNI, prognostic nutritional index; CONUT, Controlling Nutritional Status; CXI, cachexia index.

## Data Availability

As this is a systematic review, no new data were created in this study. The data presented in this review are available within the published articles cited in the references.

## References

[1] Siegel R.L., Miller K.D., Fuchs H.E., Jemal A. (2022). Cancer statistics, 2022. CA Cancer J. Clin..

[2] Antoun S., Birdsell L., Sawyer M.B., Venner P., Escudier B., Baracos V.E. (2010). Association of skeletal muscle wasting with treatment with sorafenib in patients with advanced renal cell carcinoma: Results from a placebo-controlled study. J. Clin. Oncol..

[3] Antoun S., Baracos V.E., Birdsell L., Escudier B., Sawyer M.B. (2010). Low body mass index and sarcopenia associated with dose-limiting toxicity of sorafenib in patients with renal cell carcinoma. Ann. Oncol..

[4] Fearon K., Strasser F., Anker S.D., Bosaeus I., Bruera E., Fainsinger R.L., Jatoi A., Loprinzi C., MacDonald N., Mantovani G. (2011). Definition and classification of cancer cachexia: An international consensus. Lancet Oncol..

[5] Arai H., Maeda K., Wakabayashi H., Naito T., Konishi M., Assantachai P., Auyeung W.T., Chalermsri C., Chen W., Chew J. (2023). Diagnosis and outcomes of cachexia in Asia: Working Consensus Report from the Asian Working Group for Cachexia. J. Cachexia Sarcopenia Muscle.

[6] Gu W., Wu J., Liu X., Zhang H., Shi G., Zhu Y., Ye D. (2017). Early skeletal muscle loss during target therapy is a prognostic biomarker in metastatic renal cell carcinoma patients. Sci. Rep..

[7] Fukushima H., Nakanishi Y., Kataoka M., Tobisu K.I., Koga F. (2017). Postoperative Changes in Skeletal Muscle Mass Predict Survival of Patients With Metastatic Renal Cell Carcinoma Undergoing Cytoreductive Nephrectomy. Clin. Genitourin. Cancer.

[8] Khan A.I., Psutka S.P., Patil D.H., Hong G., Williams M.A., Bilen M.A., Sekhar A., Kissick H.T., Narayan V.M., Joshi S.S. (2022). Sarcopenia and systemic inflammation are associated with decreased survival after cytoreductive nephrectomy for metastatic renal cell carcinoma. Cancer.

[9] Liu Q., Yang J., Chen X., Yang J., Zhao X., Huang Y., Lin Y., Pu J. (2022). Prognostic significance of sarcopenia and systemic inflammation for patients with renal cell carcinoma following nephrectomy. Front. Oncol..

[10] Yamashita S., Deguchi R., Hamamoto S., Bando Y., Toyoda S., Tomida R., Miyake M., Ito N., Yamaguchi N., Furukawa J. (2026). Cachexia index as a prognostic indicator in patients with metastatic renal cell carcinoma treated with nivolumab plus ipilimumab: Multicenter analysis. Int. J. Clin. Oncol..

[11] Aslan V., Kılıç A.C.K., Sütcüoğlu O., Eraslan E., Bayrak A., Öksüzoğlu B., Tahtacı G., Özdemir N., Üner A., Günel N. (2022). Cachexia index in predicting outcomes among patients receiving immune checkpoint inhibitor treatment for metastatic renal cell carcinoma. Urol. Oncol..

[12] Pretto F., Ghilardi C., Moschetta M., Bassi A., Rovida A., Scarlato V., Talamini L., Fiordaliso F., Bisighini C., Damia G. (2015). Sunitinib prevents cachexia and prolongs survival of mice bearing renal cancer by restraining STAT3 and MuRF-1 activation in muscle. Oncotarget.

[13] Piccirillo R., Giavazzi R. (2015). Inactivating STAT3: Bad for tumor, good for muscle. Cell Cycle.

[14] Ikemoto S., Sugimura K., Yoshida N., Wada S., Yamamoto K., Kishimoto T. (2000). TNF alpha, IL-1 beta and IL-6 production by peripheral blood monocytes in patients with renal cell carcinoma. Anticancer Res..

[15] Zhu W., Xu H., Xiong X., Li Y., Feng D., Huang W., Wei Q., Yang L. (2026). Decoding GDF15: Impact on prostate cancer metabolism, chemoresistance, and clinical applications. Chin. Med. J..

[16] Akdogan O., Turkmen S., Uyar G.C., Yucel K.B., Tufekci B., Gurler F., Yazici O., Ozdemir N., Ozet A., Karakaya C. (2024). Impact of Serum GDF-15 and IL-6 on Immunotherapy Response in Cancer: A Prospective Study. Cancers.

[17] Wei G., Sun H., Dong K., Hu L., Wang Q., Zhuang Q., Zhu Y., Zhang X., Shao Y., Tang H. (2021). The thermogenic activity of adjacent adipocytes fuels the progression of ccRCC and compromises anti-tumor therapeutic efficacy. Cell Metab..

[18] Tomida R., Ozaki K., Tanaka Y., Komatsu R., Hirata S., Uchida T., Furukawa J., Nikawa T., Fukawa T. (2026). Regulatory mechanisms of mitochondrial function by cancer-derived exosomes in cachexia. Front. Oncol..

[19] Ipek O.M., Dincer E., Aydiner O., Sevinc A.H., Hanci Sevinc B., Aras E. (2026). Preoperative Psoas Muscle Index and Psoas Attenuation in Patients Undergoing Nephrectomy for Renal Cell Carcinoma: A Retrospective Cohort Study. Medicina.

[20] Schmeusser B.N., Ali A.A., Fintelmann F.J., Garcia J.M., Williams G.R., Master V.A., Psutka S.P. (2023). Imaging Techniques to Determine Degree of Sarcopenia and Systemic Inflammation in Advanced Renal Cell Carcinoma. Curr. Urol. Rep..

[21] Takemura K., Yuasa T., Fujiwara R., Ito M., Suzuki H., Yonese J., Koga F. (2020). Prognostic Significance of the Controlling Nutritional Status (CONUT) Score in Patients with Advanced Renal Cell Carcinoma Treated with Nivolumab after Failure of Prior Tyrosine Kinase Inhibitors. J. Urol..

[22] Liu T., Zhou Z., Yao Y., Hu Y., Sun L., Zhang G. (2025). The Impact of Computed Tomography-Defined Sarcopenia on Surgical Outcomes in Patients with Sarcomatoid Renal Cell Carcinoma: A Comprehensive Assessment of Abdominal, Psoas, and Paraspinal Musculature. Clin. Genitourin. Cancer.

[23] Guo Z., He J., Tang G., Qiu J., Chan F.L., Wang Z., Bai Z., Wen Z. (2025). The role of preoperative sarcopenia in post-nephrectomy prognosis of renal cell carcinoma: An integrated systematic review and meta-analysis. World J. Surg. Oncol..

[24] Cushen S.J., Power D.G., Teo M.Y., MacEneaney P., Maher M.M., McDermott R., O’Sullivan K., Ryan A.M. (2017). Body Composition by Computed Tomography as a Predictor of Toxicity in Patients With Renal Cell Carcinoma Treated With Sunitinib. Am. J. Clin. Oncol..

[25] Ishihara H., Nishimura K., Ikeda T., Fukuda H., Yoshida K., Iizuka J., Kondo T., Takagi T. (2024). Impact of body composition on outcomes of immune checkpoint inhibitor combination therapy in patients with previously untreated advanced renal cell carcinoma. Urol. Oncol..

[26] Wang J., Dong P., Qu Y., Xu W., Zhou Z., Ning K., Peng Y., Xiong L., Li Z., Zou X. (2023). Association of computed tomography-based body composition with survival in metastatic renal cancer patient received immunotherapy: A multicenter, retrospective study. Eur. Radiol..

[27] Okamura M., Shirado K., Shirai N., Yagi T., Inoue T., Ogawa M., Sullivan E.S., Von Haehling S., Springer J., Anker S.D. (2026). Combined Nutritional and Exercise Interventions for Cachexia in Chronic Diseases: A Systematic Review and Meta-analysis Limited to Cancer Cachexia. Prog. Rehabil. Med..

[28] Onishi T., Ohishi Y., Iizuka N., Shirakawa H., Hatano T. (1995). A study on the relationship between the production of cytokines and the biological features of cachexia using renal cell carcinoma heterotransplanted to nude mice. Nihon Hinyokika Gakkai Zasshi.

[29] Kays J.K., Koniaris L.G., Cooper C.A., Pili R., Jiang G., Liu Y., Zimmers T.A. (2020). The Combination of Low Skeletal Muscle Mass and High Tumor Interleukin-6 Associates with Decreased Survival in Clear Cell Renal Cell Carcinoma. Cancers.

[30] Al-Lamki R.S., Mayadas T.N. (2015). TNF receptors: Signaling pathways and contribution to renal dysfunction. Kidney Int..

[31] Zhou H., Wang C., Ou C., Lao Y., Luo Y., Lao M., Liu J., Xie J., Huang H. (2026). Insulin resistance-related indices, sarcopenia, and cardiovascular risk in CKM stages 1-3: A prospective cohort study from CHARLS. BMC Endocr. Disord..

[32] Higgins M.I., Martini D.J., Patil D.H., Nabavizadeh R., Steele S., Williams M., Joshi S.S., Narayan V.M., Sekhar A., Psutka S.P. (2021). Sarcopenia and modified Glasgow Prognostic Score predict postsurgical outcomes in localized renal cell carcinoma. Cancer.

[33] Ishihara H., Kondo T., Omae K., Takagi T., Iizuka J., Kobayashi H., Tanabe K. (2016). Sarcopenia and the Modified Glasgow Prognostic Score are Significant Predictors of Survival Among Patients with Metastatic Renal Cell Carcinoma Who are Receiving First-Line Sunitinib Treatment. Target. Oncol..

[34] Remaily B.C., Young G., Lathrop H., Thomas J., Kim K., Vu T.T., Adeluola A., Stanton C., Mulcahy G., Xie Z. (2025). Tumor intrinsic properties dictate Fc receptor expression and cancer cachexia associated increase in checkpoint inhibitor clearance. Front. Immunol..

[35] Baker L.A., Wilkinson T.J., Abbott A.R., Cardoso D.F., Parker N., Denniff M.J., Billany R.E., March D.S., Young H.M., Highton P. (2026). Exploring the role of chemerin in skeletal muscle phenotype in those with kidney disease. Transl. Res..

[36] Buchler T., Kopecka M., Zemankova A., Wiesnerová M., Streckova E., Rozsypalova A., Melichar B., Poprach A., Richter I. (2020). Sarcopenia in Metastatic Renal Cell Carcinoma Patients Treated with Cabozantinib. Target. Oncol..

[37] Ishihara H., Takagi T., Kondo T., Fukuda H., Yoshida K., Iizuka J., Tanabe K. (2018). Effect of Changes in Skeletal Muscle Mass on Oncological Outcomes During First-Line Sunitinib Therapy for Metastatic Renal Cell Carcinoma. Target. Oncol..

[38] Ozkan E., Koksal M., Ece B., Koyun M., Kuzu O.F., Acikgoz Y., Algin E. (2025). Baseline and dynamic changes in skeletal muscle mass as predictive biomarkers in patients with metastatic renal cell carcinoma treated with Nivolumab. Radiol. Oncol..

[39] Hu Q., Mao W., Wu T., Xu Z., Yu J., Wang C., Chen S., Chen S., Xu B., Xu Y. (2021). High Neutrophil-to-Lymphocyte Ratio and Platelet-to-Lymphocyte Ratio Are Associated With Sarcopenia Risk in Hospitalized Renal Cell Carcinoma Patients. Front. Oncol..

[40] Gu W., Zhang G., Sun L., Ma Q., Cheng Y., Zhang H., Shi G., Zhu Y., Ye D. (2015). Nutritional screening is strongly associated with overall survival in patients treated with targeted agents for metastatic renal cell carcinoma. J. Cachexia Sarcopenia Muscle.

[41] Sun S., Zhang J. (2026). A CT-based radiomics model for noninvasive prediction of MMP9 expression and prognostic evaluation in renal cell carcinoma. Medicine.

[42] Tamir S., Behar H.V., Tal R., Jasper R.T., Armoni M., Aloni H.P., Orad R.I., Voet H., Atar E., Grubstein A. (2026). CT Body Composition Changes Predict Survival in Immunotherapy-Treated Cancer Patients: A Retrospective Cohort Study. Cancers.

[43] McManus H.D., Zhang D., Schwartz F.R., Wu Y., Infield J., Ho E., Armstrong A.J., George D.J., Kruse D., Gupta R.T. (2023). Relationship Between Pretreatment Body Composition and Clinical Outcomes in Patients With Metastatic Renal Cell Carcinoma Receiving First-Line Ipilimumab Plus Nivolumab. Clin. Genitourin. Cancer.

[44] Martin L., Hopkins J., Malietzis G., Jenkins J.T., Sawyer M.B., Brisebois R., MacLean A., Nelson G., Gramlich L., Baracos V.E. (2018). Assessment of Computed Tomography (CT)-Defined Muscle and Adipose Tissue Features in Relation to Short-Term Outcomes After Elective Surgery for Colorectal Cancer: A Multicenter Approach. Ann. Surg. Oncol..

[45] Abe K., Shibata K., Naito T., Otsuka A., Karayama M., Maekawa M., Miyake H., Suda T., Kawakami J. (2022). Impacts of cachexia progression in addition to serum IgG and blood lymphocytes on serum nivolumab in advanced cancer patients. Eur. J. Clin. Pharmacol..

[46] Rolley C., Zidane M., Nedelcu C., Barth M., Saulnier P., Procaccio V., Bigot P. (2026). Feasibility and Safety of a Ketogenic Diet During Systemic Therapy for Metastatic Renal Cell Carcinoma: Results from the Cetorein Pilot Study. Nutrients.

[47] Temel J.S., Abernethy A.P., Currow D.C., Friend J., Duus E.M., Yan Y., Fearon K.C. (2016). Anamorelin in patients with non-small-cell lung cancer and cachexia (ROMANA 1 and ROMANA 2): Results from two randomised, double-blind, phase 3 trials. Lancet Oncol..

[48] Laird B.J.A., Skipworth R., Bonomi P.D., Fallon M., Kaasa S., Giorgino R., McMillan D.C., Currow D.C. (2025). Anamorelin Efficacy in Non-Small-Cell Lung Cancer Patients With Cachexia: Insights From ROMANA 1 and ROMANA 2. J. Cachexia Sarcopenia Muscle.

[49] Nielsen R.L., Houlind M.B., Andersen A.L. (2025). Ponsegromab for the Treatment of Cancer Cachexia. N. Engl. J. Med..

[50] Emmerson P.J., Wang F., Du Y., Liu Q., Pickard R.T., Gonciarz M.D., Coskun T., Hamang M.J., Sindelar D.K., Ballman K.K. (2017). The metabolic effects of GDF15 are mediated by the orphan receptor GFRAL. Nat. Med..

[51] Frey C. (2026). GLP-1 receptor agonists and immune checkpoint inhibitor therapy: A narrative review on mechanistic and clinical evidence. Future Oncol..

[52] Albor C., Anyiam O., Boyle L.D., Makaronidis J., Tan R., Iftikhar S., Burzic A., Lajeunesse Trempe F., Stanley M., Bhatti A. (2026). The Role of GLP1 Receptor Agonists and Multi-agonist Incretin Therapies for Specific Obesity-related Health Conditions: Evidence and Rationale for Prioritisation. Curr. Obes. Rep..

[53] Pati B., Nandy N., Bindhani B.K., Bal N.C. (2026). Pro-inflammatory cytokine-driven molecular signaling in skeletal muscle during cancer cachexia. Biochem. Soc. Trans..

[54] Chen I.M., Johansen J.S., Theile S., Silverman L.M., Pelz K.R., Madsen K., Dajani O., Lim K.Z.M., Lorentzen T., Gaafer O. (2025). Randomized Phase II Study of Nab-Paclitaxel and Gemcitabine With or Without Tocilizumab as First-Line Treatment in Advanced Pancreatic Cancer: Survival and Cachexia. J. Clin. Oncol..

[55] Du Y., Liu X.Y., Pan R.L., Zhang X.T., Si X.Y., Chen M.J., Wang M.Z., Zhang L. (2024). Tocilizumab for Advanced Non-Small-Cell Lung Cancer With Concomitant Cachexia: An Observational Study. J. Cachexia Sarcopenia Muscle.

[56] Hong D.S., Hui D., Bruera E., Janku F., Naing A., Falchook G.S., Piha-Paul S., Wheler J.J., Fu S., Tsimberidou A.M. (2014). MABp1, a first-in-class true human antibody targeting interleukin-1α in refractory cancers: An open-label, phase 1 dose-escalation and expansion study. Lancet Oncol..

[57] Hickish T., Andre T., Wyrwicz L., Saunders M., Sarosiek T., Kocsis J., Nemecek R., Rogowski W., Lesniewski-Kmak K., Petruzelka L. (2017). MABp1 as a novel antibody treatment for advanced colorectal cancer: A randomised, double-blind, placebo-controlled, phase 3 study. Lancet Oncol..

[58] Cheung W.W., Zheng R., Hao S., Wang Z., Gonzalez A., Zhou P., Hoffman H.M., Mak R.H. (2021). The role of IL-1 in adipose browning and muscle wasting in CKD-associated cachexia. Sci. Rep..

